# PD-L1 is upregulated by EBV-driven LMP1 through NF-κB pathway and correlates with poor prognosis in natural killer/T-cell lymphoma

**DOI:** 10.1186/s13045-016-0341-7

**Published:** 2016-10-13

**Authors:** Xi-wen Bi, Hua Wang, Wen-wen Zhang, Jing-hua Wang, Wen-jian Liu, Zhong-jun Xia, Hui-qiang Huang, Wen-qi Jiang, Yu-jing Zhang, Liang Wang

**Affiliations:** 1 Department of Hematologic Oncology, State Key Laboratory of Oncology in South China/Cancer Center, Collaborative Innovation Center for Cancer Medicine, Sun Yat-sen University, Guangzhou, Guangdong 510060 People’s Republic of China; 2 Department of Medical Oncology, Sun Yat-sen University Cancer Center, Guangzhou, Guangdong 510060 People’s Republic of China; 3 Department of Hematologic Oncology, Sun Yat-sen University Cancer Center, Guangzhou, Guangdong 510060 People’s Republic of China; 4 Department of Radiation Oncology, Sun Yat-sen University Cancer Center, Guangzhou, Guangdong 510060 People’s Republic of China

**Keywords:** Natural killer/T-cell lymphoma, Latent membrane protein 1, Epstein–Barr virus, Programmed cell death receptor 1

## Abstract

**Background:**

Natural killer/T-cell lymphoma (NKTCL) is an Epstein–Barr virus (EBV)-associated, highly aggressive lymphoma. Treatment outcome remains sub-optimal, especially for advanced-stage or relapsed diseases. Programmed cell death receptor 1 (PD-1) and PD ligand 1 (PD-L1) have become promising therapeutic targets for various malignancies, but their role in the pathogenesis and their interactions with EBV in NKTCL remains to be investigated.

**Methods:**

Expression of PD-L1 was measured in NK-92 (EBV-negative) and SNK-6 (EBV-positive) cells by western blot, quantitative real-time PCR and enzyme-linked immunosorbent assay, and flow cytometry, respectively. Latent membrane protein 1 (LMP1)-harboring lentiviral vectors were transfected into NK-92 cells to examine the correlation between LMP1 and PD-L1 expression. Proteins in the downstream pathways of LMP1 signaling were measured in NK-92 cells transfected with LMP1-harboring or negative control vectors as well as in SNK-6 cells. PD-L1 expression on tumor specimens and serum concentration of soluble PD-L1 were collected in a retrospective cohort of patients with Ann Arbor stage I~II NKTCL, and their prognostic significance were analyzed.

**Results:**

Expression of PD-L1 was significantly higher in SNK-6 cells than in NK-92 cells, at both protein and mRNA levels. Expression of PD-L1 was remarkably upregulated in NK-92 cells transfected with LMP1-harboring lentiviral vectors compared with those transfected with negative control vectors. Proteins in the MAPK/NF-κB pathway were upregulated in LMP1-expressing NK-92 cells compared with the negative control. Selective inhibitors of those proteins induced significant downregulation of PD-L1 expression in LMP1-expressing NK-92 cells as well as in SNK-6 cells. Patients with a high concentration of serum soluble PD-L1 (≥3.4 ng/ml) or with a high percentage of PD-L1 expression in tumor specimens (≥38 %) exhibited significantly lower response rate to treatment and remarkably worse survival, compared with their counterparts. A high concentration of serum soluble PD-L1 and a high percentage of PD-L1 expression in tumor specimens were independent adverse prognostic factors among patients with stage I~II NKTCL.

**Conclusions:**

PD-L1 expression positively correlated LMP1 expression in NKTCL, which was probably mediated by the MAPK/NF-κB pathway. PD-L1 expression in serum and tumor tissues has significant prognostic value for early-stage NKTCL.

**Electronic supplementary material:**

The online version of this article (doi:10.1186/s13045-016-0341-7) contains supplementary material, which is available to authorized users.

## Background

Natural killer/T-cell lymphoma (NKTCL) is a distinct and aggressive clinicopathologic entity in the World Health Organization (WHO) classification of hematopoietic and lymphoid malignancies [1–3]. NKTCL is rare in North America and Europe but is more common in East Asia and South America [4]. This disease predominates in young males, and most cases originate from the nasal cavity [1, 2, 4]. No standard treatment strategy has been established due to the rarity of NKTCL. Radiotherapy (RT) has yielded curative effects for early-stage disease [5–7]. Anthracycline-based chemotherapeutic regimens have shown disappointing efficacy, probably due to the overexpression of multidrug-resistant genes [8–10]. Novel regimens containing l-asparaginase or pegaspargase have elicited promising responses [11–13]. However, the outcome of NKTCL remains sub-optimal, especially for advanced-stage or relapsed diseases [14, 15]. Therefore, it is in urgent need to identify novel therapeutic targets and corresponding agents.

Tumor immune escape is an emerging hallmark of cancer. Programmed cell death receptor 1 (PD-1) and PD ligand 1 (PD-L1) are important immune checkpoint molecules involved in T cell-mediated immune response and are key regulators of tumor immune escape [16–18]. Aberrant expression of PD-1/PD-L1 on tumor cells or tumor-infiltrating lymphocytes has conferred adverse prognostic impact in multiple solid and hematopoietic malignancies [19–23]. Blockade of the PD-1/PD-L1 interactions with monoclonal antibodies has achieved encouraging efficacy and has been approved by the US Food and Drug Administration (FDA) in many malignancies [24–27]. Expression of PD-L1 on tumor cells has been reported in patients with NKTCL, which may be a potential therapeutic target in the future [28, 29]. However, the role of PD-1/PD-L1 in the pathogenesis of NKTCL remains poorly understood.

There is a close correlation between Epstein–Barr virus (EBV) infection and NKTCL. Almost all cases of NKTCL exhibited a positive result of EBV-encoded RNA in situ hybridization in tumor samples. Additionally, pre- and post-treatment levels of circulating EBV DNA had significant prognostic implications for NKTCL patients [30–33]. In previous studies, expression of PD-1/PD-L1 could be upregulated by EBV infection in various malignancies [34–37], and blockade of PD-1/PD-L1 interactions successfully inhibited EBV-induced lymphoma growth in a mouse model [38], suggesting a possible interaction between EBV and PD-1/PD-L1 pathway in tumor immunology. Whether such an interaction exists in NKTCL, a typical EBV-associated malignancy, remains to be explored. In the present study, we aim to explore the interaction between EBV infection and PD-L1 expression in NKTCL cell lines and the clinical significance of PD-L1 expression in NKTCL patients.

## Methods

### Cell lines and culture

The SNK-6 and NK-92 cells were routinely kept in Sun Yat-sen University Cancer Center (SYSUCC) and were incubated in a humidified incubator with 5 % CO_2_ at 37 °C. The SNK-6 cell line was cultured in RPMI-1640 (Gibco, USA) medium containing 2 mmol/l glutamine, 100 U/ml penicillin, and 100 μg/ml streptomycin, supplemented with 1000 U/ml interleukin (IL)-2 (Sigma-Aldrich, USA) and 10 % human AB serum (Gemini Bioproducts, Woodland, CA, USA). The NK-92 cells were maintained in α-MEM (Life Technologies, Karlsruhe, Germany) containing 20 % FBS (Gibco, USA), 2 mM l-glutamate, 100 mg/ml penicillin, and 100 mg/ml streptomycin (Life Technologies) and supplemented with 10 ng/ml IL-2 (Sigma-Aldrich, USA).

### Western blot analysis

Cells were washed with ice-cold PBS and were suspended in radioimmunoprecipitation assay (RIPA) lysis buffer (Biyuntian Biotech, Shanghai, China) containing 1 % phenylmethylsulfonyl. After centrifugation at 14,000 rpm for 10 min at 4 °C, the protein content of supernatant was determined using the Pierce™ BCA Protein Assay Kit (Thermo Scientific, Rockford, IL, USA). Aliquots (20 μg protein per lane) were separated by 12 % sodium dodecyl sulfate polyacrylamide gel electrophoresis (SDS-PAGE) and transferred to polyvinylidene fluoride (PVDF) membranes (Millipore, Billerica, MA, USA). The membranes were exposed to primary antibodies and glyceraldehyde-3-phosphate dehydrogenase (GAPDH) (1: 2000, Abcam) at room temperature for 1 h, followed by labeling with horseradish peroxidase-conjugated goat anti-rabbit IgG (1: 20,000, Boster, Wuhan, China) for 40 min at room temperature. Signals were detected with enhanced chemiluminescence plus reagents (Amersham Pharmacia, Piscataway, NJ, USA). GAPDH was used as the internal control. The following primary antibodies were used: PD-L1, B-Raf, p-B-Raf, p38, p-p38, ERK, pERK (Abcam, Shanghai, China), latent membrane protein 1 (LMP1), JNK, pJNK (Santa Cruz, Shanghai, China), and p65 (Boster, Wuhan, China).

### Quantitative real-time PCR (qRT-PCR) analysis

In order to quantify PD-L1 and LMP1 mRNA, total RNA was isolated from SNK-6 and NK-92 cells using TRIZOL Reagent (Invitrogen, USA) according to the instruction manual. One microgram of the total RNA was reversely transcribed into cDNA using Bestar™ qPCR RT Kit (DBI Bioscience, China). The qRT-PCR reaction was prepared in a total volume of 20 μl containing 10 μl DBI Bestar® SybrGreen qPCR Master Mix (DBI Bioscience, China), cDNA derived from 0.2 μg of input RNA, 5 pM each primer, and 7 μl double-distilled H_2_O. The PCR was run on Stratagene Mx3000P Real-Time PCR system (Agilent Technologies, USA). The fluorescent quantity PCR conditions were as follows: pre-denaturation at 95 °C for 2 min, followed by 40 cycles of 94 °C for 20 s, 58 °C for 20 s, and 72 °C for 30 s. Primers were as follows: PD-L1 forward 5′-GAACTACCTCTGGCACATCCT-3′, PD-L1 reverse 5′-CACATCCATCATTCTCCCTTT-3′; LMP1 forward 5′-CAACAACGGCAAGACTCCC-3′, LMP1 reverse 5′-CCTCAAAGAAGCCACCCTC-3′. Each reaction was replicated three times. The fold changes in cDNA relative to the GAPDH endogenous control were calculated using the 2^−ΔΔCt^ method [39].

### Measurement of soluble PD-L1 in cell culture supernatant

The cell mixture was centrifuged at 1500 rpm for 5 min. The supernatant was collected and determined for the concentration of soluble PD-L1 using a sandwich enzyme-linked immunosorbent assay (ELISA) kit (PDCD1LG1 ELISA kit, Cloud-Clone Corp., Wuhan, China) according to the manufacturer’s instructions.

### Flow cytometry analysis

Cells were labeled with anti-PD-L1 antibody (Alexa Fluor 647; Abcam, Shanghai, China) and then were assayed by flow cytometry using the Cytomics FC 500MPL cytometer. Data were collected and analyzed with the CXP version 2.2 software (Beckman Coulter Inc.).

### Construction of a LMP1-expressing NK-92 cell line

The LMP1-expressing lentivirus vector, LV5-LMP1, was constructed by the insertion of a full-length LMP1 cDNA into LV5 vector (GenePharma Co. Ltd., China) at *NotI* and *BamHI* sites. The LV5-LMP1 vector and LV5 control vector (LV5-NC) were, respectively, cotransfected with packaging vectors pGag/Pol, pRev, and pVSV-G (GenePharma Co. Ltd., China) into HEK-293T cells using Lipofectamine 2000 Transfection Reagent (Beyotime, Shanghai, China). After culturing for 72 h, the supernatants of the transfected cells were harvested. The lentiviral titers were determined by flow cytometric analysis for green fluorescence protein (GFP) expressed by viral vectors. 1 × 10^5^/well NK-92 cells were infected with LV5-LMP1 and LV5-NC vectors, respectively, at a multiplicity of infection (MOI) of 300. After culturing for 48 h, western blot and ELISA were performed to determine the expression of proteins in NK-92 cells infected by LV5-LMP1 and LV5-NC, respectively.

### Measurement of serum soluble PD-L1 in NKTCL patients

Serum samples were collected before treatment from 77 patients with newly diagnosed NKTCL between 2008 and 2015 at SYSUCC and from 15 healthy volunteers. Serum was collected from the whole blood by centrifuging at 4000×*g* and stored at −80 °C. The level of soluble PD-L1 was determined using a sandwich ELISA kit (PDCD1LG1 ELISA kit, Cloud-Clone Corp., Wuhan, China) as per the manufacturer’s protocol.

### Immunohistochemical analysis of PD-L1 in biopsy specimen from NKTCL patients

Paraffin-embedded specimens were collected before treatment from the same NKTCL patients described above. Four-micrometer-thick sections were deparaffinized, rehydrated, and quenched. Immunohistochemical staining was performed using an anti-PD-L1 rabbit polyclonal antibody (1:50 dilution, Abcam, Cambridge, UK) and a two-stage immunohistochemical kit (ChemMate™ Envision Detection Kit, Peroxidase/DAB, Dako, Glostrup, Denmark) according to the manufacturer’s instructions. The number of all tumor cells and those with membrane PD-L1 staining were calculated manually under high magnification (×200) using Image Pro Plus 6.0 software (Media Cybernetics, Maryland, USA). Seven fields were calculated for each individual specimen to determine the percentage of tumor cells with membrane staining among all tumor cells. In order to minimize intra-tumor heterogeneity, two fields with the highest and lowest percentages were eliminated, and the average percentage of the remaining five fields was used to represent the level of PD-L1 expression for an individual.

### Clinical data and treatment

Seventy-seven patients with previously untreated NKTCL diagnosed at SYSUCC between 2008 and 2015 were enrolled in this study. The diagnosis was based on the WHO classification of hematopoietic and lymphoid tumors, and all patients had positive results for EBV-encoded RNA (EBER) in situ hybridization (ISH) [1, 2]. The clinical characteristics and treatment modalities are summarized in Table 1. All patients had Ann Arbor stage I or II disease. The International Prognostic Index (IPI) and the natural killer/T-cell lymphoma prognostic index (NKPI) were calculated for risk stratification [40, 41]. The majority of patients were categorized into the low-risk IPI (0–1, 89.6 %) or NKPI (0–1, 72.7 %) group. All patients received induction chemotherapy followed by consolidative radiotherapy (RT) as their primary treatment. 66.2 % of the patients received GELOX (gemcitabine, l-asparaginase, and oxaliplatin) and 33.8 % received CHOP-L (cyclophosphamide, doxorubicin, vincristine, prednisone, and l-asparaginase) as induction chemotherapeutic regimen in dosages as previously reported [42]. Complete remission (CR) was achieved after primary therapy in 76.6 % of the patients. The median follow-up period for this cohort was 38.0 (9.4–79.0) months.

**Table 1:** The clinical characteristics and treatment modalities of patients with NK/T-cell lymphoma

Parameters	Total *n* (%)	PD-L1 < 3.4 ng/ml *n* (%)	PD-L1 ≥ 3.4 ng/ml *n* (%)	*P* value	PD-L1 < 38 % *n* (%)	PD-L1 ≥ 38 % *n* (%)	*P* value
Overall	77 (100)	51 (100)	26 (100)	–	51 (100)	26 (100)	–
Male gender	42 (54.5)	23 (45.1)	19 (73.1)	0.020	26 (51.0)	16 (61.5)	0.379
Age > 60 years	11 (14.3)	10 (19.6)	1 (3.8)	0.087	9 (17.6)	2 (7.7)	0.316
ECOG score ≥ 2	4 (5.2)	1 (2.0)	3 (11.5)	0.109	2 (3.9)	2 (7.7)	0.600
Ann Arbor stage							
I	41 (53.2)	31 (60.8)	10 (38.5)	0.063	30 (58.8)	11 (42.3)	0.170
II	36 (46.8)	20 (39.2)	16 (61.5)		21 (41.2)	15 (57.7)	
B symptoms	22 (28.6)	12 (23.5)	10 (38.5)	0.170	13 (25.5)	9 (34.6)	0.433
LDH > 245 U/L	18 (23.4)	10 (19.6)	8 (30.8)	0.274	10 (19.6)	8 (30.8)	
IPI score							
0–1	69 (89.6)	47 (92.2)	22 (84.6)	0.432	47 (92.2)	22 (84.6)	0.432
2	8 (10.4)	4 (7.8)	4 (15.4)		4 (7.8)	4 (15.4)	
NKPI score							
0–1	56 (72.7)	39 (76.5)	17 (65.4)	0.302	38 (74.5)	18 (69.2)	0.623
2–3	21 (27.3)	12 (23.5)	9 (34.6)		13 (25.5)	8 (30.8)	
Chemotherapy regimen							
GELOX	51 (66.2)	34 (66.7)	17 (65.4)	0.910	35 (68.6)	16 (61.5)	0.534
CHOP-L	26 (33.8)	17 (33.3)	9 (34.6)		16 (31.4)	10 (38.5)	
Treatment response							
CR	59 (76.6)	43 (84.3)	16 (61.5)	0.026	43 (84.3)	16 (61.5)	0.026
Non-CR	18 (23.4)	8 (15.7)	10 (38.5)		8 (15.7)	10 (38.5)	

*Abbreviations: CR* complete remission, *ECOG* Eastern Cooperative Oncology Group, *IPI* International Prognostic Index, *LDH* lactate dehydrogenase, *NKPI* natural killer/T-cell lymphoma prognostic index

### Statistical analysis

Continuous variables were compared using a two-tailed Student’s *t* test, and categorical variables were compared using the chi-square test or the Fisher’s exact test. Overall survival (OS) was measured from the date of diagnosis to the date of death or the most recent follow-up. Progression-free survival (PFS) was measured from the date of diagnosis to the date of disease progression, death, or the most recent follow-up. Survival data were calculated with the Kaplan-Meier method and compared using the log-rank test. Variables with statistical significance in univariate analysis were included in the multivariate analysis using a stepwise forward Cox regression model. Optimal cut-off values of serum and histological PD-L1 levels for predicting survival were determined using the receiver operating characteristics (ROC) curve analysis. The Spearman correlation test was used to explore the correlation between the serum and histological PD-L1 levels. Differences were considered statistically significant with a two-sided *P* value of <0.05. The statistical analysis was performed using SPSS version 17.0 software (SPSS, Inc., Chicago, IL, USA).

## Results

### PD-L1 expression was higher in EBV+NKTCL cell line

**Fig. 1:**
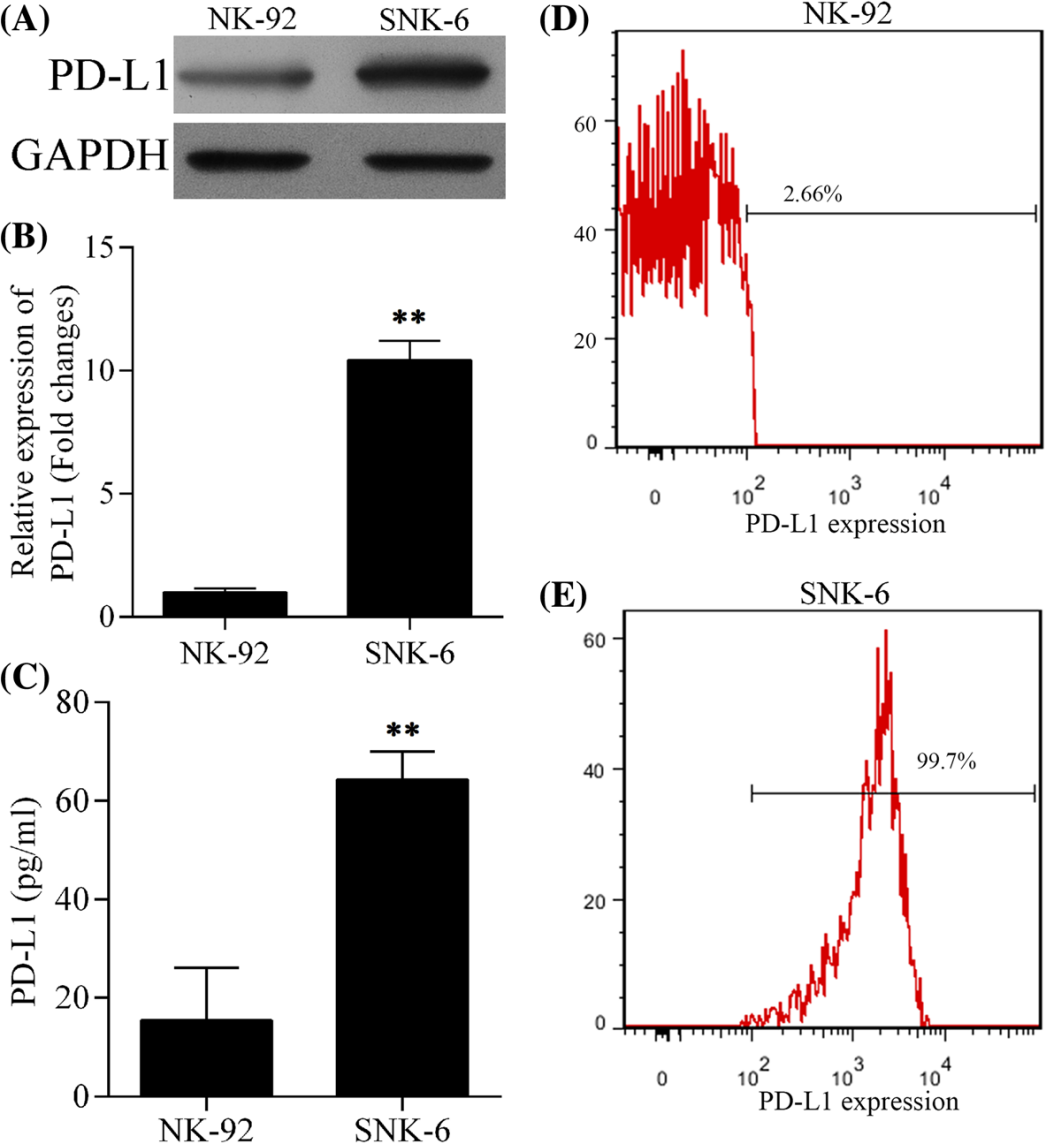
Expression of PD-L1 in NK cell line NK-92 (EBV-negative) and NKTCL cell line SNK-6 (EBV-positive). The level of **a** PD-L1 protein detected by western blot, **b** PD-L1 mRNA detected by quantitative real-time PCR, **c** soluble PD-L1 protein in cell culture supernatant detected by ELISA, and **d**, **e** PD-L1 expression on cell surface detected by flow cytometry in NK-92 and SNK-6 cell lines, respectively. ^**^ *P* < 0.05

Western blot, ELISA, flow cytometry, and qRT-PCR were performed to determine protein and mRNA levels of PD-L1, respectively, in two cell lines: the human NK cell line NK-92 (EBV-negative) and the NKTCL cell line SNK-6 (EBV-positive). The protein level of PD-L1 was remarkably higher in SNK-6 cell line than that in NK-92 cells (Fig. 1a). Consistently, the relative expression level of PD-L1 mRNA in SNK-6 cells was also significantly higher than that in NK-92 cells (Fig. 1b, *P* < 0.05). ELISA of cell culture supernatants found a remarkably higher level of soluble PD-L1 produced by SNK-6 cells than NK-92 cells (Fig. 1c, *P* < 0.05). Additionally, expression of PD-L1 on cell surface was much higher in SNK-6 cells (Fig. 1e) than in NK-92 cells (Fig. 1d).

### LMP1 upregulated PD-L1 expression in NK-92 cells

**Fig. 2:**
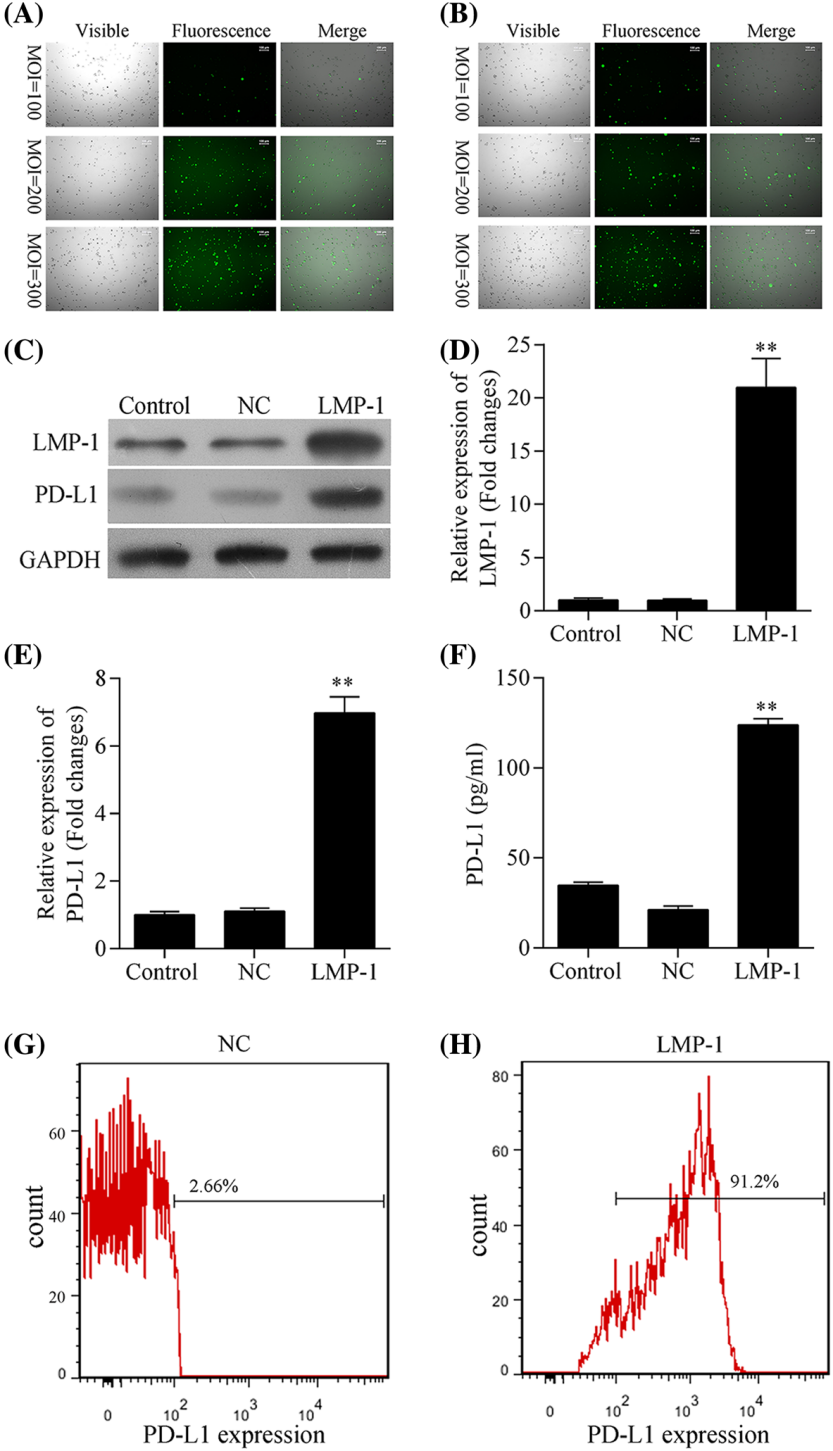
Expression of PD-L1 was upregulated by LMP1 in NK-92 cells. **a**, **b** Infection efficiency of LV5-LMP1 vector (**a**) and LV5 vector (LV5-NC, **b**) in NK-92 cell line at a multiplicity of infection (MOI) of 100, 200, and 300, respectively. The level of **c** LMP1 and PD-L1 proteins detected by western blot, **d** LMP1 and **e** PD-L1 mRNA detected by quantitative real-time PCR, **f** soluble PD-L1 protein in cell culture supernatant detected by ELISA, and **g**, **h** PD-L1 expression on cell surface detected by flow cytometry in LMP1-expressing NK92 (LMP1) and negative control NK92 (NC) cell lines, respectively. ^**^ *P* < 0.05

To investigate whether LMP1 expression in NK-92 cells was associated with upregulated PD-L1 level, we constructed a novel LMP1-expressing NK-92 cell line using a LV5-LMP1 lentiviral vector. Good infection efficiency of both LV5-LMP1 and LV5-NC vectors for NK-92 cell lines was observed at an MOI of 300 (Fig. 2a, b). Western blot and qRT-PCR found remarkably higher levels of LMP1 protein (Fig. 2c) and mRNA (Fig. 2d, *P* < 0.05) in NK-92 cells transfected with LV5-LMP1 vector, compared with those transfected with LV5-NC. Accordingly, remarkable upregulation of both PD-L1 protein (Fig. 2c) and mRNA (Fig. 2e, *P* < 0.05) was observed in the LMP1-expressing NK-92 cells compared with the negative control. ELISA found a significantly higher concentration of soluble PD-L1 in cell supernatants produced by LMP1-expressing NK-92 compared with the negative control (Fig. 2f, *P* < 0.05). Additionally, expression of PD-L1 on cell surface was much higher in LMP1-expressing NK-92 cells (Fig. 2h) than in the negative control (Fig. 2g).

### LMP1 upregulated PD-L1 expression through MAPK/NF-κB pathway in NK-92 cells

**Fig. 3:**
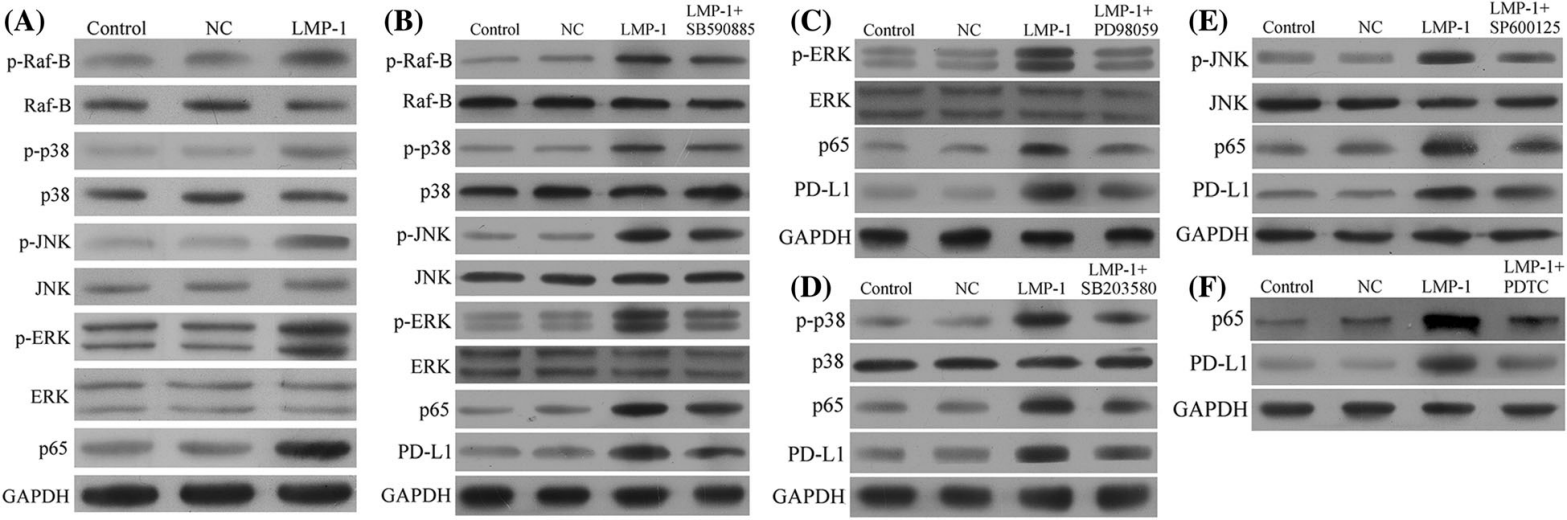
LMP1 upregulated PD-L1 expression through MAPK/NF-κB pathway in NK92 cells. **a** The expression level of p-Raf-B, Raf-B, p-p38, p38, p-JNK, JNK, p-ERK, ERK, and p65 in LMP1-expressing NK92 (LMP1) and negative control NK92 (NC) cell lines. **b** The expression level of p-Raf-B, Raf-B, p-p38, p38, p-JNK, JNK, p-ERK, ERK, p65, and PD-L1 in negative control NK92 (NC) or LMP1-expressing NK92 (LMP1) cell line treated with 0.1 μM SB590885, a selective B-Raf inhibitor for 1 h. **c** The expression level of p-ERK, ERK, p65, and PD-L1 in negative control NK92 (NC) or LMP1-expressing NK92 (LMP1) cell line treated with 20 μM PD98059, a selective ERK inhibitor for 1 h. **d** The expression level of p-p38, p38, p65, and PD-L1 in negative control NK92 (NC) or LMP1-expressing NK92 (LMP1) cell line treated with 10 μM SB203580, a selective p38 inhibitor for 1 h. **e** The expression level of p-JNK, JNK, p65, and PD-L1 in negative control NK92 (NC) or LMP1-expressing NK92 (LMP1) cell line treated with 20 μM SP600125, a selective JNK inhibitor for 1 h. **f** The expression level of p65 and PD-L1 in negative control NK92 (NC) or LMP1-expressing NK92 (LMP1) cell line treated with 100 μM pyrrolidine dithiocarbamate (PDTC), a selective inhibitor of NF-κB for 1 h

To explore the possible underlying mechanisms of PD-L1 upregulation induced by LMP1, we examined the expression of several proteins in the downstream pathways of LMP1. As shown in Fig. 3a, p-Raf-B, p-p38, p-JNK, p-ERK, and p65 were upregulated in the LMP1-expressing NK-92 cells compared with the negative control. A selective inhibitor of B-Raf (SB590885) effectively inhibited the expression of p-Raf-B, p-ERK, and p65 in LMP1-expressing NK-92 cells, resulting in reduced PD-L1 expression (Fig. 3b). Additionally, PD98059 (an ERK inhibitor), SB203580 (a p38 inhibitor), and SP600125 (a JNK inhibitor) could all effectively suppress the expression of p65 as well as PD-L1 (Fig. 3c–e). Finally, pyrrolidine dithiocarbamate (PDTC) as a selective inhibitor of NF-κB could remarkably reduce the expression of PD-L1 in LMP1-expressing NK-92 cells (Fig. 3f). These data suggested that MAPK/NF-κB pathway may be responsible for the LMP1-induced PD-L1 expression in human NK-92 cells. In addition, we further repeated the experiments above in SNK-6 cells. The results demonstrated that the effects of lowered PD-L1 expression seen in LMP1-expressing NK-92 cell line by B-Raf, ERK, p38, JNK, or NF-κB inhibition could also be observed in SNK-6 cells, which confirmed the observations made in the transfected cell line (see Additional file 1: Figure S1).

### Pretreatment histological PD-L1 expression and serum soluble PD-L1 concentration correlated with survival in early-stage NKTCL patients

**Fig. 4:**
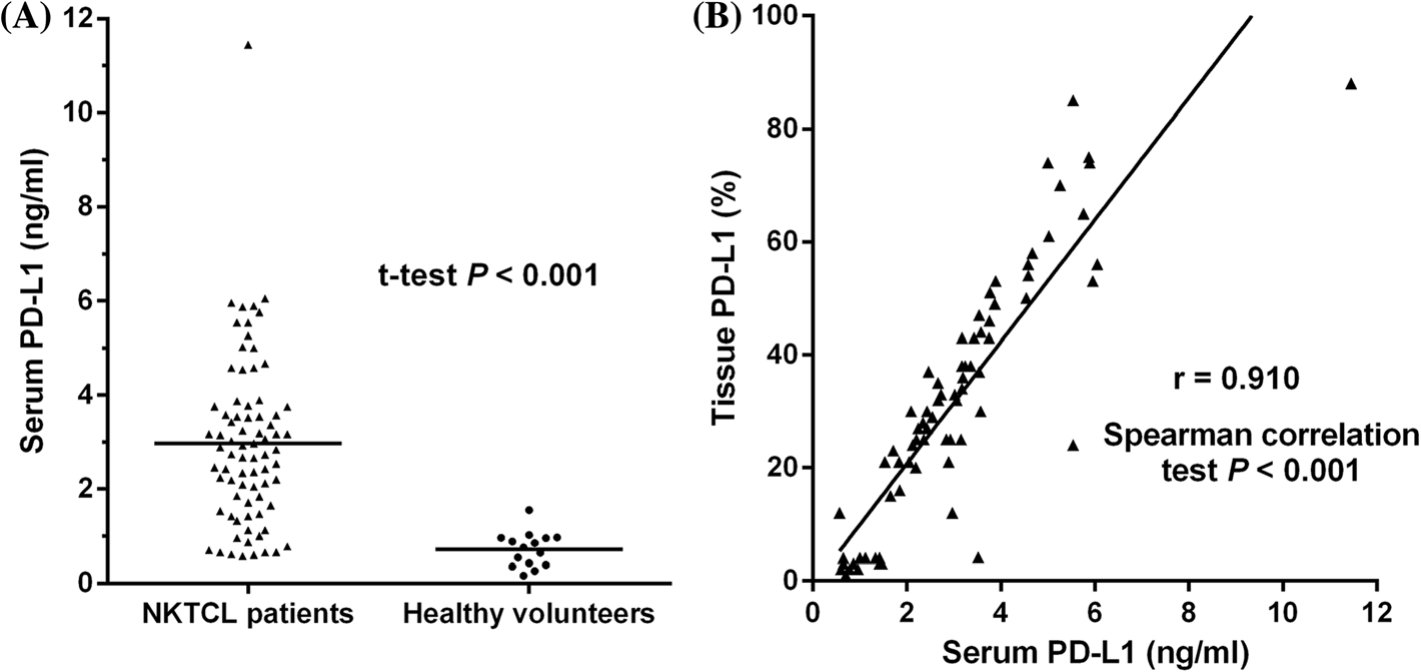
**a** Concentration of serum soluble PD-L1 in patients with natural killer/T-cell lymphoma (NKTCL) and healthy individuals. **b** Correlation between concentration of serum soluble PD-L1 (ng/ml) and PD-L1 expression in tumor tissues (%) in patients with NKTCL

To explore the clinical significance of PD-L1 in NKTCL, we retrospectively examined the level of PD-L1 expression in paraffin-embedded tissues as well as the serum concentration of soluble PD-L1 in 77 patients with stage I–II disease. As shown in Fig. 4b, the expression level of PD-L1 in tumor tissues positively correlated with pretreatment serum concentration of soluble PD-L1 (*P* < 0.001). Patients with NKTCL had a significantly higher concentration of serum soluble PD-L1 than healthy individuals (Fig. 4a, *P* < 0.001).

**Fig. 5:**
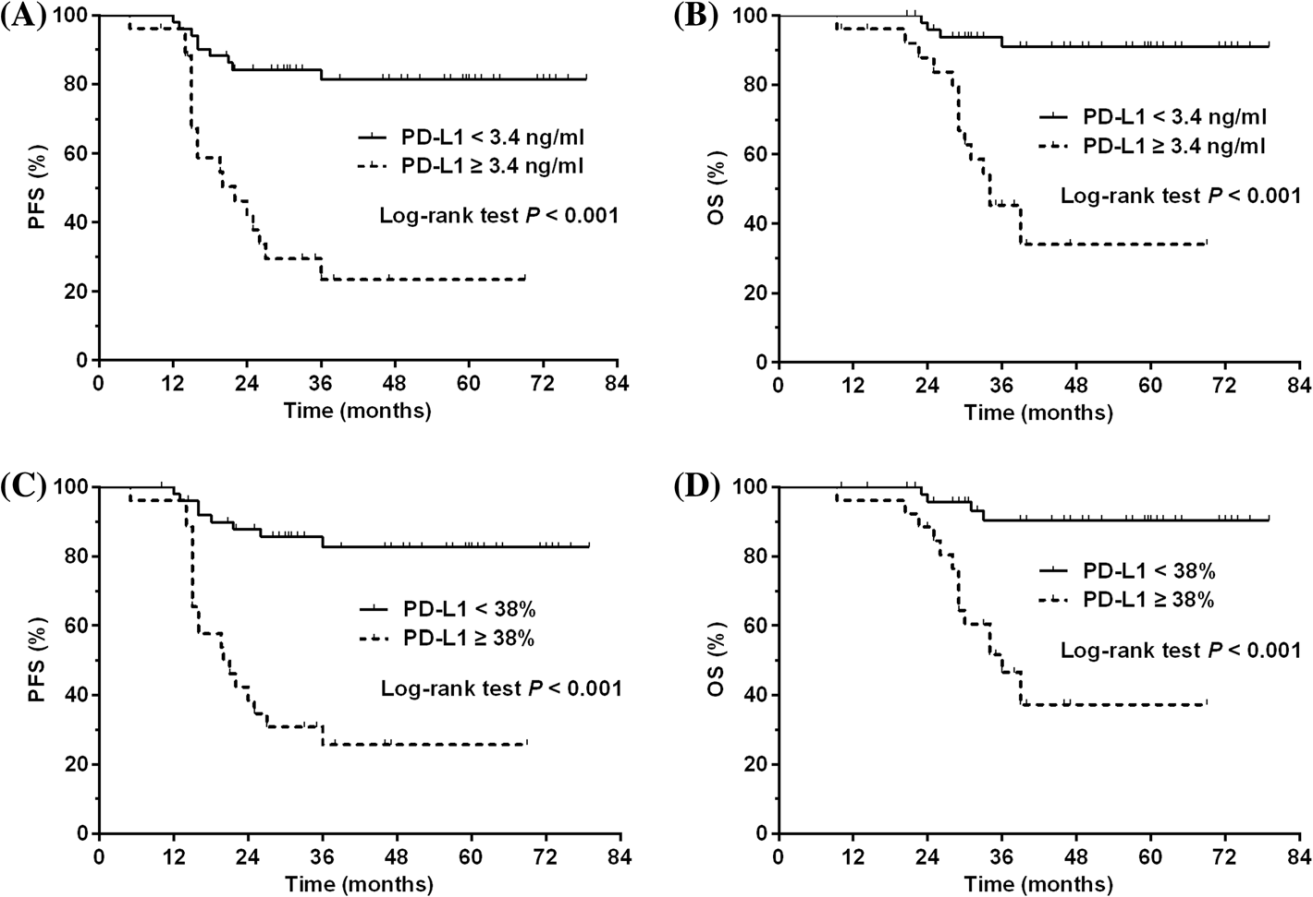
**a** Progression-free survival (PFS) for NKTCL patients with a serum soluble PD-L1 of <3.4 and ≥3.4 ng/ml. **b** Overall survival (OS) for NKTCL patients with a serum soluble PD-L1 of <3.4 and ≥3.4 ng/ml. **c** PFS for NKTCL patients with a histological PD-L1 expression of <38 and ≥38 %. **c** OS for NKTCL patients with a histological PD-L1 expression of <38 and ≥38 %

The optimal cut-off value of serum PD-L1 was 3.4 ng/ml based on the ROC analysis (see Additional file 2: Figure S2A). As shown in Table 1, clinical characteristics were almost comparable between patients with low (<3.4 ng/ml) or high (≥3.4 ng/ml) concentration of serum PD-L1, except a male predominance observed in patients with high levels of serum PD-L1 (73.1 vs. 45.1 %, *P* = 0.020). After primary therapy, patients with a serum PD-L1 ≥ 3.4 ng/ml achieved a significantly lower CR rate than those with a serum PD-L1 < 3.4 ng/ml (61.5 vs. 84.3 %, *P* = 0.026, Table 1). Univariate analysis showed a significantly inferior survival in patients with a serum PD-L1 ≥ 3.4 ng/ml compared with those with a serum PD-L1 < 3.4 ng/ml (3-year PFS: 23.5 vs. 81.4 %, *P* < 0.001, Fig. 5a; 3-year OS: 45.3 vs. 91.0 %, *P* < 0.001, Fig. 5b). Serum PD-L1 ≥ 3.4 ng/ml remained an independent adverse prognostic factor for PFS and OS in multivariate analysis (Table 2).

**Table 2:** Univariate and multivariate analyses of prognostic factors in patients with NK/T-cell lymphoma (model 1)

Variable	Progression-free survival			Overall survival		
	Univariate analysis	Multivariate analysis		Univariate analysis	Multivariate analysis	
	*P* value	HR (95 % CI)	*P* value	*P* value	HR (95 % CI)	*P* value
Gender (female vs. male)	0.025			0.010		
Age (>60 vs. ≤60 years)	0.660			0.887		
ECOG score (≥2 vs. 0–1)	0.124			0.015		
Stage (II vs. I)	0.001	3.06 (1.30–7.22)	0.010	0.003	4.49 (1.34–15.02)	0.015
B symptoms (yes vs. no)	0.251			0.264		
LDH (elevated vs. normal)	0.020			0.001		
IPI (2 vs. 0–1)	0.006			0.001	3.29 (1.13–9.57)	0.029
NKPI (2–3 vs. 0–1)	0.095			0.058		
Chemotherapy (GELOX vs. CHOP-L)	0.661			0.800		
Treatment response (non-CR vs. CR)	0.001	3.50 (1.53–8.01)	0.003	0.001	5.99 (1.89–18.98)	0.002
Serum PD-L1 (≥3.4 vs. <3.4 ng/ml)	<0.001	4.98 (2.22–11.21)	<0.001	<0.001	6.76 (2.12–21.59)	0.001

*Abbreviations: CI* confidence interval, *CR* complete remission, *ECOG* Eastern Cooperative Oncology Group, *HR* hazard ratio, *IPI* International Prognostic Index, *LDH* lactate dehydrogenase, *NKPI* natural killer/T-cell lymphoma prognostic index

Representative images for PD-L1 staining in tumor tissues are shown in Additional file 3: Figure S3. The optimal cut-off value of histological PD-L1 expression in tumor tissues was 38 % based on the ROC analysis (see Additional file 2: Figure S2B). Histological PD-L1 expression was not associated with clinical characteristics (Table 1). A significantly lower rate of CR after treatment was observed in patients with PD-L1 expression of ≥38 % compared with those with PD-L1 expression of <38 % (61.5 vs. 84.3 %, *P* = 0.026, Table 1). Univariate analysis showed a significantly worse survival in patients with a PD-L1 expression of ≥38 % compared with those with a PD-L1 expression of <38 % (3-year PFS 25.6 vs. 82.6 %, *P* < 0.001, Fig. 5c; 3-year OS 46.5 vs. 90.4 %, *P* < 0.001, Fig. 5d). Multivariate analysis found a PD-L1 expression of ≥38 % an independent adverse prognostic factor for PFS and OS (Table 3).

**Table 3:** Univariate and multivariate analyses of prognostic factors in patients with NK/T-cell lymphoma (model 2)

Variable	Progression-free survival			Overall survival		
	Univariate analysis	Multivariate analysis		Univariate analysis	Multivariate analysis	
	*P* value	HR (95 % CI)	*P* value	*P* value	HR (95 % CI)	*P* value
Gender (female vs. male)	0.025	2.61 (1.13–6.02)	0.025	0.010		
Age (>60 vs. ≤60 years)	0.660			0.887		
ECOG score (≥2 vs. 0–1)	0.124			0.015		
Stage (II vs. I)	0.001			0.003		
B symptoms (yes vs. no)	0.251			0.264		
LDH (elevated vs. normal)	0.020	2.26 (1.01–5.07)	0.048	0.001		
IPI (2 vs. 0–1)	0.006			0.001	4.29 (1.48–12.41)	0.007
NKPI (2–3 vs. 0–1)	0.095			0.058		
Chemotherapy (GELOX vs. CHOP-L)	0.661			0.800		
Treatment response (non-CR vs. CR)	0.001			0.001		
Histological PD-L1 (≥38 vs. <38 %)	<0.001	6.76 (2.91–15.69)	<0.001	<0.001	7.34 (2.36–22.86)	0.001

*Abbreviations: CI* confidence interval, *CR* complete remission, *ECOG* Eastern Cooperative Oncology Group, *HR* hazard ratio, *IPI* International Prognostic Index, *LDH* lactate dehydrogenase, *NKPI* natural killer/T-cell lymphoma prognostic index

## Discussion

Due to the relative rarity, sub-optimal current treatment strategies, and commonly observed chemoresistance of NKTCL, it is urgently warranted to identify novel therapeutic targets. Blockade of PD-1/PD-L1 interactions has emerged as a promising immunotherapy for cancer patients [24–27]. Previous studies have revealed aberrant expressions of PD-1/PD-L1 in NKTCL cell lines and tissues as well as involvement of PD-1/PD-L1 in the downregulation of antitumor immunity, suggesting that PD-1/PD-L1 may serve as a potential candidate for immunotherapy in NKTCL [28]. In the present study, we focused on the interactions between EBV infection and PD-L1 expression in NKTCL cell lines, as well as the prognostic impact of PD-L1 expression in NKTCL patients. Our findings included the following: (1) PD-L1 expression positively correlated LMP1 expression at both protein and mRNA levels in NKTCL and NK cells; (2) PD-L1 expression was upregulated by LMP1 through the MAPK/NF-κB pathway; and (3) the levels of PD-L1 expression on tumor tissues and the pre-treatment concentration of serum soluble PD-L1 correlated with the survival in early-stage NKTCL patients treated with asparaginase-containing chemotherapy combined with RT.

EBV plays a pivotal role in the pathogenesis of several hematopoietic malignancies [43]. It has been reported that overexpression of PD-L1 are commonly observed in EBV-associated lymphomas, including classical Hodgkin’s lymphoma, EBV-positive diffuse large B-cell lymphoma, angioimmunoblastic T-cell lymphoma, and NKTCL [28, 29, 43, 44]. Recent in vitro studies have found that LMP1, an EBV-encoded antigen, was able to upregulate PD-L1 expression in EBV-associated malignancies, such as Hodgkin’s lymphoma, post-transplant lymphoproliferative disorders, and nasopharyngeal carcinoma [37, 45]. In agreement with those results, we also observed a significant upregulation of PD-L1 expression at both protein and mRNA levels induced by LMP1 expression in NKTCL. First, the PD-L1 expression was much higher in EBV-positive SNK-6 cells than in the EBV-negative NK-92 cells. Additionally, the induction of LMP1 expression in NK-92 cells with a lentiviral vector resulted in remarkable elevations of PD-L1 protein and mRNA. Our findings imply that EBV infection in NKTCL probably upregulate PD-L1 expression on tumor cells via LMP1 antigen, and therefore induce immune tolerance.

The underlying mechanisms of PD-1/PD-L1 activation vary between different types of cancers [45–47]. Activation of the NF-κB pathway has been observed in NKTCL and is involved in proliferation, invasiveness, metastasis, and chemoresistance [48, 49]. LMP1 has been reported to contribute to the aberrant activation of the NF-κB pathway in NKTCL [50, 51]. In addition, previous studies using genome-wide miRNA expression profiling and exome sequencing revealed upregulation of the MAPK signaling pathway in NKTCL, but its biological significance remains to be understood [52, 53]. In the present study, the results showed a correlation between LMP1 and upregulation of PD-L1 expression in NK-92 cells, and the MAPK and NF-κB pathways were potentially involved. In line with our findings, Fang et al. reported that LMP1 upregulated PD-L1 through STAT3, MAPKs/AP-1, and NF-κB pathways in nasopharyngeal carcinoma [45]. The results mutually show that MAPK and NF-κB pathways may be potentially responsible for the LMP1-induced upregulation of PD-L1 expression in EBV-driven malignancies.

In a retrospective cohort of 30 NKTCL patients reported by Han et al., PD-L1 was aberrantly expressed in nasal NKTCL specimens compared with the rhinitis specimens and PD-L1 expression closely correlated with some clinical and histopathological parameters [28]. However, the prognostic impact of PD-L1 expression in NKTCL patients was not reported in this study. Another finding of our study is that the levels of PD-L1 expression on tumor tissues and the concentration of serum soluble PD-L1 correlated with survival in early-stage NKTCL patients. For early-stage (stage I~II) NKTCL, RT has been well established as the primary treatment [5–7], and chemotherapy may yield additional benefits for high-risk individuals [54]. Chemotherapies containing asparaginase (such as GELOX regimen) have produced superior response than anthracycline-based regimens (such as CHOP or EPOCH regimen) [8–13]. In our previous study, patients with early-stage NKTCL achieved a 3-year OS of 87.0 % after receiving GELOX chemotherapy plus RT, which was significantly better than that in patients receiving CHOP (54.0 %) or EPOCH (54.0 %) plus RT [42]. In the present study, early-stage NKTCL patients with a high concentration of serum soluble PD-L1 or a high percentage of PD-L1 expression on tumor tissues exhibited dismal survivals (3-year OS 45.3 and 46.5 %, respectively) even if they were uniformly treated with asparaginase-containing chemotherapy plus RT. Therefore, overexpression of PD-L1 confers a negative effect on survival for early-stage NKTCL and novel agents or treatment strategies are warranted for this particular subgroup of patients. Monoclonal antibodies blocking the PD-1/PD-L1 interactions have exhibited promising response in several types of lymphoma [25, 55]. There is an ongoing phase II study evaluating the efficacy and safety of pembrolizumab (a PD-1 antibody) in patients with relapsed/refractory T-cell lymphomas including NKTCL (NCT02535247), and its results may help offering another treatment choice for this relatively rare malignancy.

Several limitations exist in this retrospective study. The dynamic alterations of serum PD-L1 concentration after treatment and during follow-up, as well as their value in predicting relapse or prognosis, were not analyzed. Additionally, we did not analyze the prognostic impact of PD-L1 among patients with advanced-stage NKTCL due to the heterogeneous treatment delivered to those patients. Furthermore, we were unable to examine the levels of EBV mRNA and LMP1 expression in tumor samples due to the limited availability of paraffin-embedded tissues, which may provide useful information to understand the correlation between PD-L1 expression and EBV infection. Another issue that needs to be addressed is that the criteria of determining PD-L1 positivity varied among different studies. In Han’s study, the product of staining intensity and percentage of positive tumor cells was used to classify positive cases. However, in the present and some previous studies, only the percentage of cells with PD-L1 staining was used due to the heterogeneity of staining intensity, subjectivity of visional grading, and clinical feasibility [56–58]. A uniform and widely accepted standard to determine PD-L1 positivity is required for studies in the future.

## Conclusions

The present study revealed a positive correlation between LMP1 and PD-L1 expression, which was probably mediated by the MAPK/NF-κB pathway in NKTCL. It also showed a significant prognostic value of PD-L1 expression level on tumor tissues and serum soluble PD-L1 concentration in early-stage NKTCL. Further studies are warranted to validate our findings in a prospective cohort and to explore the therapeutic value of PD-1/PD-L1 in NKTCL.

## Additional files

Additional file 1: Figure S1. PD-L1 was upregulated in SNK-6 cells through MAPK/NF-κB pathway. (A) The expression level of p-Raf-B, Raf-B, p-p38, p38, p-JNK, JNK, p-ERK, ERK, p65, and PD-L1 in SNK-6 cells or SNK-6 cells treated with 0.1 μM SB590885, a selective B-Raf inhibitor for 1 h. (B) The expression level of p-ERK, ERK, p65, and PD-L1 in SNK-6 cells or SNK-6 cells treated with 20 μM PD98059, a selective ERK inhibitor for 1 h. (C) The expression level of p-p38, p38, p65, and PD-L1 in SNK-6 cells or SNK-6 cells treated with 10 μM SB203580, a selective p38 inhibitor for 1 h. (D) The expression level of p-JNK, JNK, p65, and PD-L1 in SNK-6 cells or SNK-6 cells treated with 20 μM SP600125, a selective JNK inhibitor for 1 h. (E) The expression level of p65 and PD-L1 in SNK-6 cells or SNK-6 cells treated with 100 μM pyrrolidine dithiocarbamate (PDTC), a selective inhibitor of NF-κB for 1 h. (TIF 1305 kb)
Additional file 2: Figure S2. The receiver operating curve (ROC) of (A) pretreatment serum concentration of PD-L1 and (B) histological PD-L1 expression in tumor tissues. The optimal cut-off value for pretreatment serum concentration of PD-L1 to predict mortality is 3.4 ng/ml (area under curve = 0.799, sensitivity = 88.9 %, specificity = 72.9 %). The optimal cut-off value for histological PD-L1 expression in tumor tissues to predict mortality is 38 % (area under curve = 0.760, sensitivity = 77.8 %, specificity = 79.7 %). (TIF 873 kb)
Additional file 3: Figure S3. Immunohistochemical analysis of PD-L1 expression in tumor tissues from patients with natural killer/T-cell lymphoma. Representative images of (A) strong and (B) weak cell membrane staining (brown) of PD-L1 are shown (×200 magnification). (TIF 7689 kb)
