# Supplementary figures and images for: PD-L1 is upregulated by EBV-driven LMP1 through NF-κB pathway and correlates with poor prognosis in natural killer/T-cell lymphoma
Source: J Hematol Oncol. 2016 Oct 13;9:109. doi: 10.1186/s13045-016-0341-7 (PMC5064887; doi:10.1186/s13045-016-0341-7)

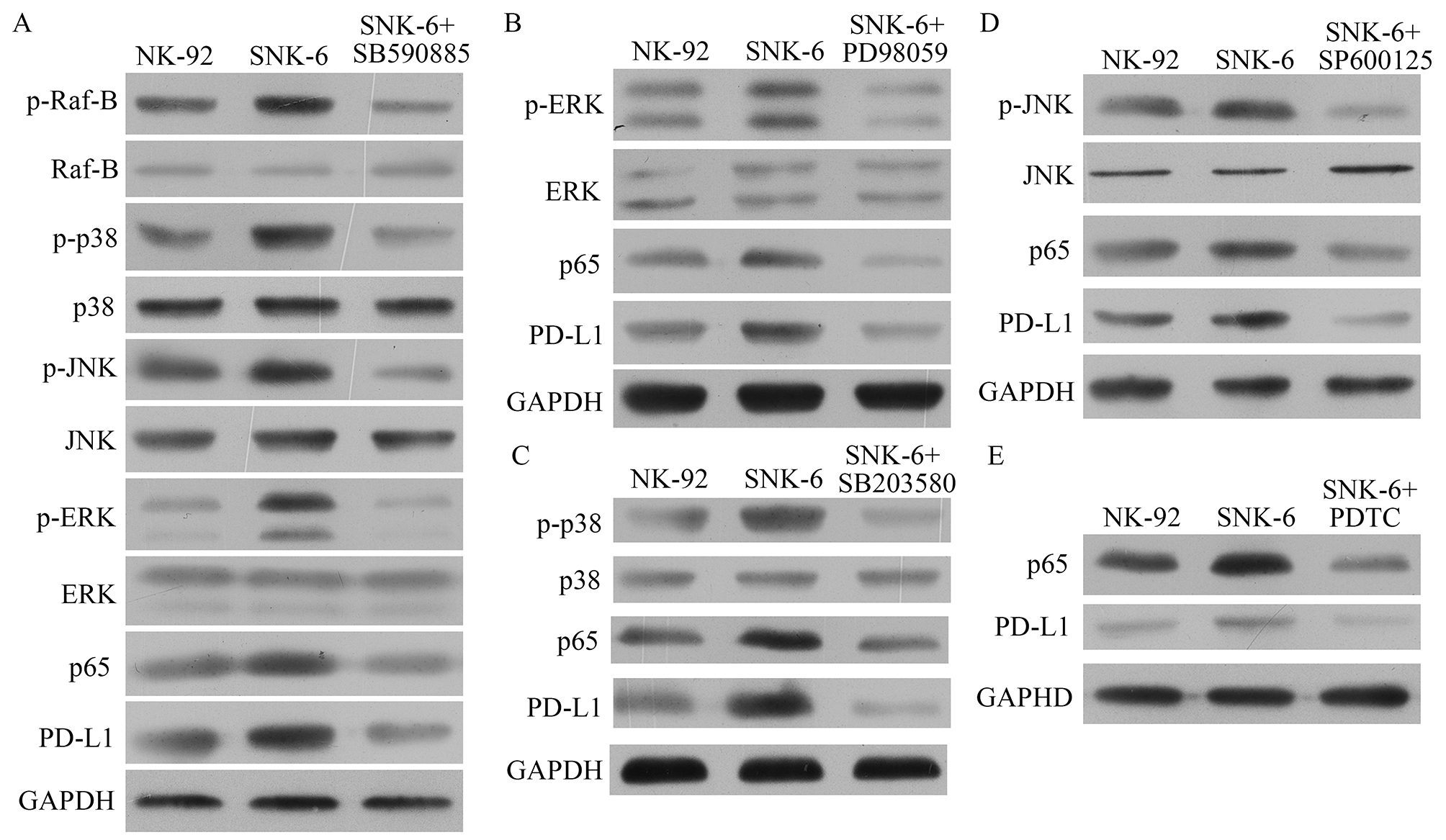

Supplement: Additional file 1: Figure S1. — PD-L1 was upregulated in SNK-6 cells through MAPK/NF-κB pathway. (A) The expression level of p-Raf-B, Raf-B, p-p38, p38, p-JNK, JNK, p-ERK, ERK, p65, and PD-L1 in SNK-6 cells or SNK-6 cells treated with 0.1 μM SB590885, a selective B-Raf inhibitor for 1 h. (B) The expression level of p-ERK, ERK, p65, and PD-L1 in SNK-6 cells or SNK-6 cells treated with 20 μM PD98059, a selective ERK inhibitor for 1 h. (C) The expression level of p-p38, p38, p65, and PD-L1 in SNK-6 cells or SNK-6 cells treated with 10 μM SB203580, a selective p38 inhibitor for 1 h. (D) The expression level of p-JNK, JNK, p65, and PD-L1 in SNK-6 cells or SNK-6 cells treated with 20 μM SP600125, a selective JNK inhibitor for 1 h. (E) The expression level of p65 and PD-L1 in SNK-6 cells or SNK-6 cells treated with 100 μM pyrrolidine dithiocarbamate (PDTC), a selective inhibitor of NF-κB for 1 h. (TIF 1305 kb) [file 13045_2016_341_MOESM1_ESM.tif]

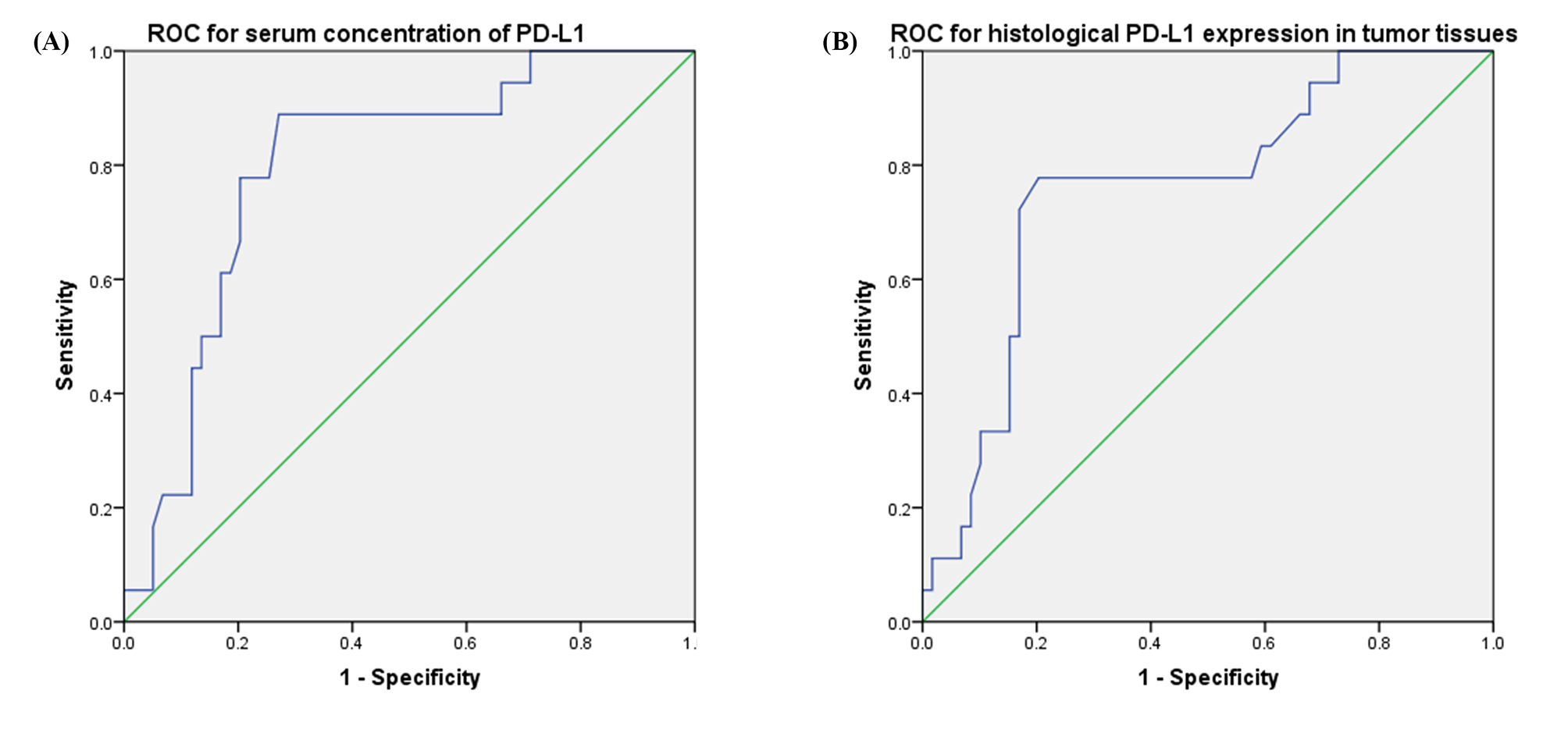

Supplement: Additional file 2: Figure S2. — The receiver operating curve (ROC) of (A) pretreatment serum concentration of PD-L1 and (B) histological PD-L1 expression in tumor tissues. The optimal cut-off value for pretreatment serum concentration of PD-L1 to predict mortality is 3.4 ng/ml (area under curve = 0.799, sensitivity = 88.9 %, specificity = 72.9 %). The optimal cut-off value for histological PD-L1 expression in tumor tissues to predict mortality is 38 % (area under curve = 0.760, sensitivity = 77.8 %, specificity = 79.7 %). (TIF 873 kb) [file 13045_2016_341_MOESM2_ESM.tif]

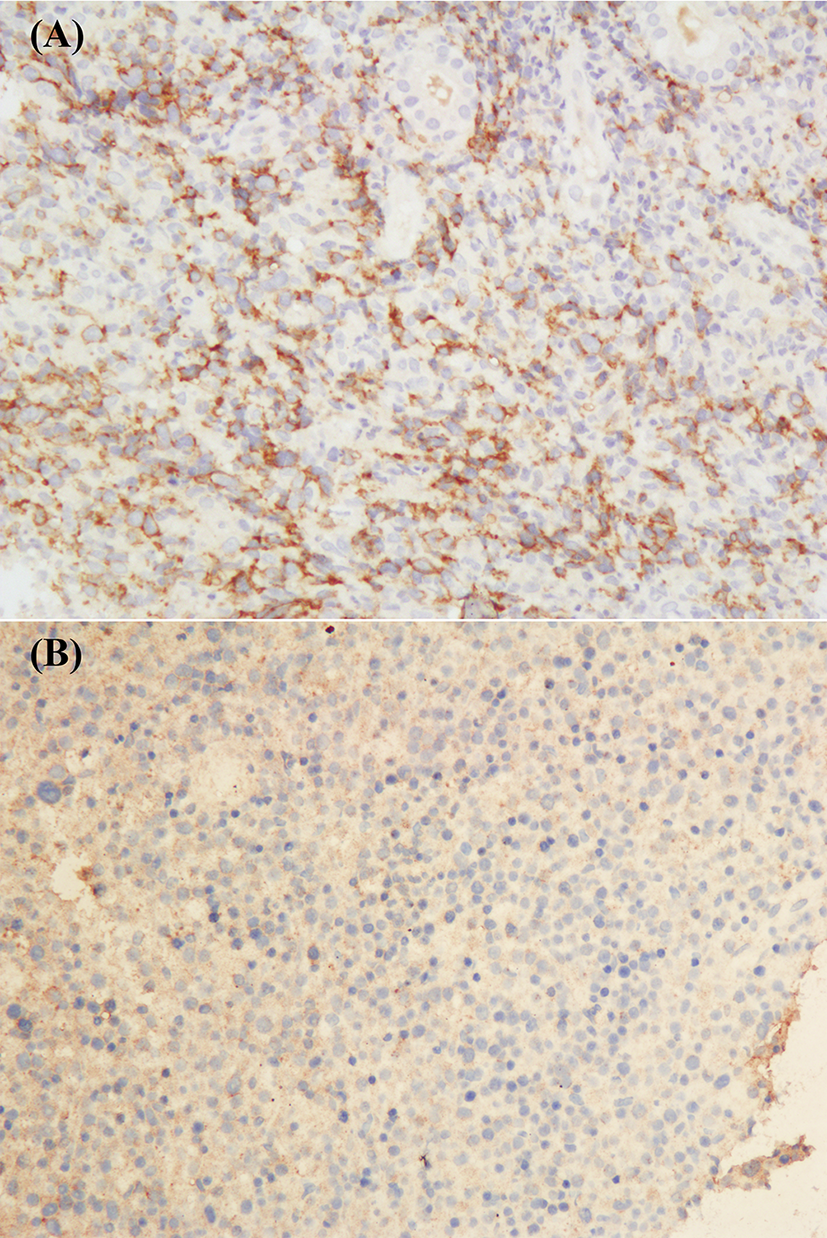

Supplement: Additional file 3: Figure S3. — Immunohistochemical analysis of PD-L1 expression in tumor tissues from patients with natural killer/T-cell lymphoma. Representative images of (A) strong and (B) weak cell membrane staining (brown) of PD-L1 are shown (×200 magnification). (TIF 7689 kb) [file 13045_2016_341_MOESM3_ESM.tif]
